# A Dynamic Stochastic Model of Frequency-Dependent Stress Fiber Alignment Induced by Cyclic Stretch

**DOI:** 10.1371/journal.pone.0004853

**Published:** 2009-03-25

**Authors:** Hui-Ju Hsu, Chin-Fu Lee, Roland Kaunas

**Affiliations:** Department of Biomedical Engineering, Texas A&M University, College Station, Texas, United States of America; CRG - Centre for Genomic Regulation, Universitat Pompeu Fabra, Spain

## Abstract

**Background:**

Actin stress fibers (SFs) are mechanosensitive structural elements that respond to forces to affect cell morphology, migration, signal transduction and cell function. Cells are internally stressed so that SFs are extended beyond their unloaded lengths, and SFs tend to self-adjust to an equilibrium level of extension. While there is much evidence that cells reorganize their SFs in response to matrix deformations, it is unclear how cells and their SFs determine their specific response to particular spatiotemporal changes in the matrix.

**Methodology/Principal Findings:**

Bovine aortic endothelial cells were subjected to cyclic uniaxial stretch over a range of frequencies to quantify the rate and extent of stress fiber alignment. At a frequency of 1 Hz, SFs predominantly oriented perpendicular to stretch, while at 0.1 Hz the extent of SF alignment was markedly reduced and at 0.01 Hz there was no alignment at all. The results were interpreted using a simple kinematic model of SF networks in which the dynamic response depended on the rates of matrix stretching, SF turnover, and SF self-adjustment of extension. For these cells, the model predicted a threshold frequency of 0.01 Hz below which SFs no longer respond to matrix stretch, and a saturation frequency of 1 Hz above which no additional SF alignment would occur. The model also accurately described the dependence of SF alignment on matrix stretch magnitude.

**Conclusions:**

The dynamic stochastic model was capable of describing SF reorganization in response to diverse temporal and spatial patterns of stretch. The model predicted that at high frequencies, SFs preferentially disassembled in the direction of stretch and achieved a new equilibrium by accumulating in the direction of lowest stretch. At low stretch frequencies, SFs self-adjusted to dissipate the effects of matrix stretch. Thus, SF turnover and self-adjustment are each important mechanisms that cells use to maintain mechanical homeostasis.

## Introduction

Actin stress fibers (SFs) are bundles of actin filaments crosslinked by α-actinin and myosin II in non-muscle cells. Large ventral SFs are anchored to the substrate at each end via focal adhesions [1], [2]. Consequently, myosin-generated contraction leads to the development of isometric tension. This tension extends SFs beyond their unloaded lengths [3], [4]. In human aortic ECs, the level of SF extension is maintained at a set-point value of approximately 1.10 [4]. SFs are dynamic structures and their continuous assembly and disassembly is critical to cellular functions involving changes in cell shape, including migration [5] and cell alignment [6]. It follows then that SFs must be constantly self-adjusting in order to maintain a set-point level of extension. Importantly, cyclic mechanical stretching of the matrix can perturb SF extension, leading to compensatory responses such as gradual alignment of SFs perpendicular to the principal direction of stretch [7], [8]. The cellular response to cyclic stretch also depends on the pattern of mechanical force applied in a manner that involves SF alignment [9], [10]. Thus, it is important to understand how cells respond to different patterns of mechanical force to regulate SF organization and cell function.

Mathematical models help to elucidate the complex relationships between matrix deformation and SF dynamics. A model put forth by De et al. [11], [12] is based on the premise that cells subjected to stretch seek to maintain constant either the local stress or strain in the surrounding matrix by adjusting a force dipole that characterizes the contractile force in highly polarized, elongated cells. Their model proposes characteristic time constants that describe the rate cells can self-adjust the magnitude and direction of the force dipole in response to cyclic stretch. When the rate of stretch is low, the model predicts that cells will readjust their contractility, resulting in alignment of the cells parallel to the direction of stretch. When the rate of stretch is sufficiently fast, the cells cannot respond quickly enough and must orient away from the direction of stretch to compensate. These predictions provide valuable insight into how cells sense and respond to dynamic stretch patterns, yet the physiological mechanism for cell reorientation is unclear. We have developed a mathematical model in which SF alignment perpendicular to the stretch direction occurs as a consequence of the accelerated disassembly of SFs whose level of extension is perturbed from a set-point level [13]. The organization of SFs in ECs subjected to cyclic uniaxial and equibiaxial stretches were well described using this model. Building upon our previous model, the present study evaluates the respective roles of the rates of SF turnover and self-adjustment as mechanisms to modulate the response of SF networks to different frequencies and magnitudes of cyclic stretch. Experiments were performed to quantify the frequency-dependence of stretch-induced SF alignment. Our modeling results indicate that SF self-adjustment determines the frequency dependency, while SF turnover determines the maximum extent of the SF orientation possible at high stretch frequencies.

## Materials and Methods

### Constrained Mixture Model of SF Networks

Let us model the stress fibers in individual cells as a mixture of coexisting, load-bearing fibers constrained to deform together as the cell deforms [13]. Following the Rule of Mixtures approach [14], each SF acts independently of the others. Given that well-spread cells are generally very flat in areas other than the peri-nuclear region and that SFs in non-muscle cells are typically localized at the ventral surface [15], we assume a two-dimensional SF network immediately adjacent to the matrix surface.

Each SF is anchored to the matrix at each end via focal adhesions, with the distance between adhesions being the current length of the SF (*l*). SFs are under tension due to myosin-generated isometric contraction. We define the unloaded length of the SF (*l*_0_) as the length of the SF if it were allowed to elastically retract to a state of zero tension (e.g., by dislodging one or both focal adhesions) [3], [16]. Lu et al. [4] reported that the level of fiber prestretch (*α_0_* = *l*/*l*_0_) in endothelial cells is approximately 1.10 and has little intracellular and intercellular variance. Consequently, we assume that all newly assembled fibers are uniformly prestretched to a magnitude *α_0_* = 1.10. The newly assembled SFs are subject to deformation in response to stretching of the extracellular matrix. For the current study, we will consider cyclic matrix deformations that follow a sinusoidal pattern. The smoothly changing matrix stretch pattern is approximated using finite elasticity theory as a series of incremental stretches applied over small time increments (Δ*t* = 0.01 s). Each incremental stretch is described by the right Cauchy-Green tensor (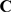) defined relative the reference configuration, which in this case is the configuration at the beginning of the time increment. In particular, 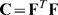 where 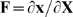 is the deformation gradient tensor. Note that any temporal pattern of cyclic stretch can be simulated using this approach by varying the magnitude of each step to match the rate of stretch at a particular point in the cycle.

Following the Rule of Mixtures, let us assume the SF network moves in registry with the matrix. Further, let us assume the fibers are only subjected to normal matrix strains, which changes the distance between focal adhesions, and hence, changes the lengths of the associated stress fibers. The deformation gradient for the *i*th SF at time *t*+Δ*t*, relative to its configuration at time *t*, is 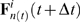, which is associated with mapping the points from the previous configuration *n*(t) to the new configuration *n*(*t*+Δ*t*). The stretch ratio of the *i^th^* SF, relative to the previous configuration is
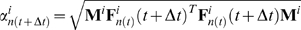
(1)where **M** *^i^* is the unit vector in the direction of the *i*th SF in its configuration at time *t*. Thus, the total stretch in the *i*th SF is at time *t*+Δ*t* is 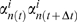.

### Stochastic Description of SF Turnover and Self-adjustment

SFs are constantly assembling and disassembling at rates dependent on their mechanical loading. Releasing the prestretch in SFs or excessively stretching SFs increases the rate of their disassembly [4]. We have previously described the dynamics of SF disassembly with a deterministic model using first-order reaction kinetics in which the rate parameter for disassembly depended on the deviation of SF stretch from the prestretch value [13]. The deterministic approach was limited by the need to discretize both the possible orientations and reference configurations for the fibers. To relax these assumptions, let us employ a stochastic approach in which the fate of each individual fiber is tracked over time.

First, let us consider the initial conditions. In a population of unstretched ECs, there is no preferred direction for the stress fibers [17]. Consequently, each cell is assumed to contain a distribution of fiber orientations where each orientation is randomly chosen from a uniform distribution between 0 and 180°. A nonuniform distribution (e.g. a von Mises distribution) can be assigned to represent more elongated cell phenotypes (e.g. fibroblasts) if necessary.

Obviously, an individual SF exists until it disassembles. Let the probability that an individual SF (existing at time *t*) will disassemble at time *t*+Δ*t* depend on the rate parameter *k* ^i^,
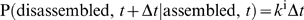
(2)where the rate parameter *k* ^i^ is dependent on the difference between the current (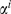) and set-point levels (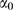) of stretch of the *i*th SF:
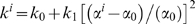
(3)The numerical integrations were performed using a time increment Δ*t* of 0.01 s. We found that decreasing Δ*t* below 0.01 s did not significantly change the results of the simulations. The total number (*N*) of SFs in the simulations was 1000. In test cases, we found that increasing *N* reduced the noise in the circular variance curves, but the system response was otherwise identical.

### Self-adjustment of SF Extension

Cells tend to maintain constant the level of pre-extension in SFs following a perturbation in stretch [4]. Kumar et al. [18] measured the gradual retraction of the severed ends of a SF subjected to laser ablation and described the rate of retraction using a viscoelastic-type function. Motivated by these results, let us describe the gradual return of SF stretch ratio to the equilibrium level following a perturbation as
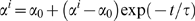
(4)where *τ* is the characteristic time for fiber stretch to return to the equilibrium value following a perturbation of magnitude 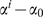.

### Cell Culture

Bovine aortic ECs (Lonza, Walkersville, MD) were cultured in GIBCO DMEM (Invitrogen) supplemented with 10% fetal bovine serum, 2 mM L-glutamine, 1 mM sodium pyruvate and 1 mM penicillin/streptomycin as previously described [17]. Cell cultures and stretch experiments were performed in a humidified 5% CO_2_-95% air incubator at 37°C.

### Stretch Experiments

ECs were subjected to cyclic stretch using a custom-built device capable of applying sinusoidally-varying stretch of different magnitudes (0–20%), frequencies (0.01–1 Hz), and patterns (e.g., pure uniaxial and equibiaxial) within the central 16 cm^2^ region of the culture chamber containing a silicone rubber membrane (Specialty Manufacturing, Saginaw, MI) [9], [17]. The central region of the membrane was coated with 10 µg/ml fibronectin (Sigma) overnight and washed with sterile PBS. ECs were cultured on this surface at low densities (100 cells/mm^2^) to avoid cell-cell contact.

### Quantification of SF Organization

Following a stretch experiment, the cells were rinsed with PBS at 37°C, fixed in 4% paraformaldehyde in PBS for 10 min at room temperature, and permeabilized with 0.5% Triton X-100 in PBS for 15 min. Actin filaments were then labeled with Alexa 488 phalloidin (Invitrogen) for 45 min at 1∶200 dilution in PBS. Images were capture with a FN1 microscope (Nikon) equipped with a DIGITAL ECLIPSE C1 plus scanning confocal head (Nikon) illuminated with a 40-mW Argon ion laser (Nikon). The images were post-processed using a custom-made algorithm in MATLAB (the MathWorks, Natick, MA). The algorithm determines pixel intensity gradients to quantify local orientations of SFs in each image [9], [13], [17]. To characterize the dispersion in SF orientations, we computed the circular variance by vectorially summing the individual orientation vector components, normalizing the result by the total number of vectors (*N_V_*) and subtracting the obtained number from unity,
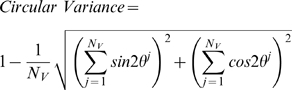
(5)where *θ^j^* is the angle for vector *j*. The values range from zero to unity, corresponding to perfectly aligned and totally uniform distributions, respectively. For determining the circular variance of a population of cells, the distribution in SF orientations was computed by summing the distributions from individual cells, with the histogram from each cell normalized so that each cell contributed equally to the total distribution. A similar circular variance was determined from the computed stress fiber distributions in the simulated cases.

## Results

### Dependence of SF alignment on stretch frequency

**Figure 1:**
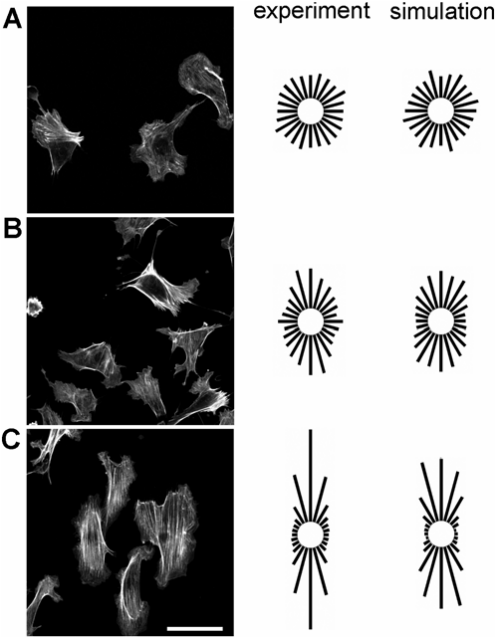
The extent of stress fiber alignment depends on the frequency of cyclic uniaxial stretch. Representative images are shown of sparsely seeded bovine aortic ECs that were subjected to 4 hr of 10% cyclic uniaxial stretch at frequencies of 1 (A), 0.1 (B), and 0.01 Hz (C), fixed, and stained for F-actin. The distributions of stress fiber orientations were determined using an intensity gradient algorithm and the results from multiple cells (n = 50 cells) are summarized as angular histograms (direction of stretch is horizontal with respect to the page). Simulations of SF reorganization in response to 4 hr of 10% cyclic uniaxial stretch at frequencies of 1, 0.1 and 0.01 Hz were performed using the optimized parameter values (*k*_0_ = 3.0×10^−4^ s^−1^, *k*_1_ = 1.8×10^4^ s^−1^, and *τ* = 0.5 s) and the angular histograms are shown for comparison to the experimental results. Bar, 50 µm.

Non-confluent bovine aortic ECs were subjected to 10% cyclic sinusoidally-varying uniaxial stretch at 0.01, 0.1, and 1 Hz and the resulting distributions in SF orientations were quantified. There was a complete lack of stress fiber alignment at 0.01 Hz (Figure 1A). At 0.1 and 1 Hz, the SFs oriented perpendicularly, with the extent of alignment noticeably higher for 1 Hz (Figures 1B and C).

### Parameter estimation

**Figure 2:**
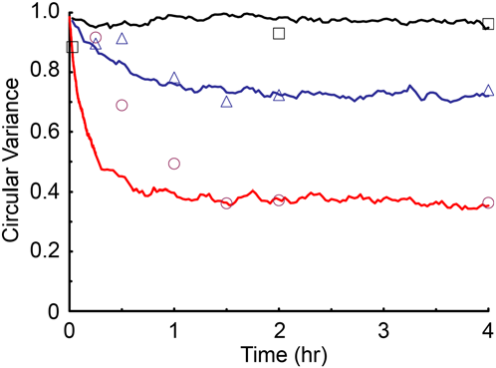
Parameter estimation using the time courses of stress fiber alignment. Circular variances of the stress fiber distributions were plotted over the period indicated to show the time courses of stress fiber alignment in response to 10% cyclic uniaxial stretch at frequencies of 1 (red circles), 0.1 (blue triangles), and 0.01 Hz (black squares). Results from simulations using the optimized parameter values (*k*_0_ = 3.0×10^−4^ s^−1^, *k*_1_ = 1.8×10^4^ s^−1^, and *τ* = 0.5 s) are illustrated for these conditions.

Simulations were performed of 10% cyclic sinusoidally-varying uniaxial stretch at 0.01, 0.1, and 1 Hz to describe the experimental data. Specifically, the time evolution of circular variance was used to estimate the model parameters (Figure 2). At 0.01 Hz, the measured circular variances remained near 0.95. For 0.1 and 1 Hz, the circular variances gradually dropped during the first 2 hours of stretch before reaching a steady state. The data were then used to extract the model parameters (*τ* = 0.5 s, *k*_0_ = 3.0×10^−4^ s^−1^ and *k*_1_ = 1.7×10^4^ s^−1^). Simulations for 10% cyclic uniaxial stretch at 0.01, 0.1 and 1 Hz illustrate that the model describes the SF distributions (Figure 1) and the time courses of alignment (Figure 2) for the experimental data reasonably well, although the model predicted that alignment occurs more quickly at 1 Hz than we observed experimentally. It is worth noting that a model with elastic SFs (i.e. *τ* →∞) is incapable of fitting all three set of data. These results illustrate the significant role of SF self-adjustment in stretch-induced SF alignment.

### Parameter Sensitivity Analysis

**Figure 3:**
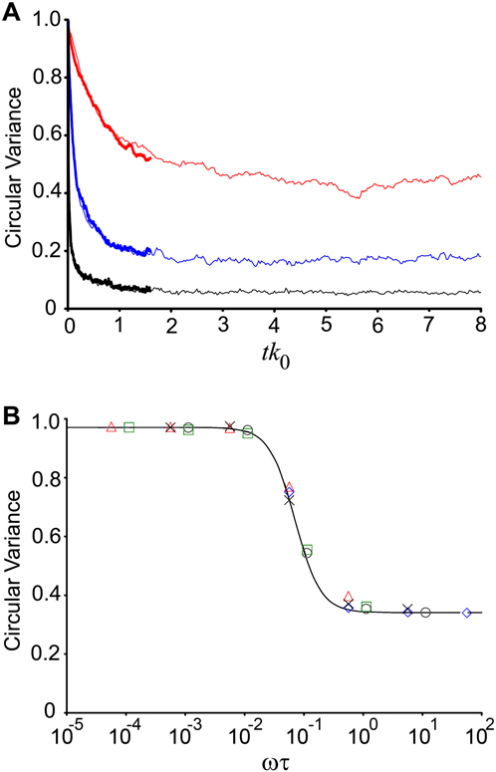
Sensitivity of the system behavior to the values of the model parameters. A: Simulations of 10% cyclic uniaxial stretch at 1 Hz were performed over a range of values for *k_0_* values of 10^−4^ (thick lines) and 10^−5^ s^−1^ (thin lines), and *k_1_* values of 10^3^ (red lines), 10^4^ (blue lines), and 10^5^ (black lines). Circular variance was plotted versus non-dimensionalized time *tk*_0_ to illustrate that the rate of alignment scales with *k*_0_, while the steady-state response depends on *k*_1_. B: The effects stretch frequency on the steady-state average circular variance are shown for of *τ* values of 0.05 (triangles), 0.1 (squares), 0.5 (crossmarks), 1 (circles) and 5 s (diamonds), with *k_0_* and *k_1_* held constant at the optimized values. Plotting circular variance versus non-dimensionalized frequency illustrates that the values for the threshold and saturation frequencies scale with *τ*.

To understand the effects of each model parameter on the system response, we performed a sensitivity analysis. The effects of varying *k*_0_ and *k*_1_ are illustrated in Figure 3A for simulations of 10% uniaxial stretch at 1 Hz. How quickly the system responds depends on *k*_0_, so we show the response in terms of a scaled time *tk*_0_. Indeed, for this scaled plot the curves for a given value of *k*_1_ and different values of *k*_0_ (10^−5^ to 10^−4^ s^−1^) form a single curve. For a given value of *k*_0_, increasing the value of *k*_1_ results in a decrease in the equilibrium value for circular variance. Thus *k*_0_ determines the rate of SF alignment, while *k*_1_ determines the extent of SF alignment.

To illustrate the effects of the rate of SF self-adjustment, we varied the value of the time constant *τ* while the other two parameters were fixed at their optimal values (Figure 3B). The relationship between the steady-state circular variance and scaled frequency (ωτ) were all described by a single curve for *τ* values ranging from 0.05 to 5 s. Below a threshold value of scaled frequency of 0.005, SFs do not become significantly oriented in response to cyclic stretch. Above a scaled frequency of ∼0.5 the circular variance at steady state reaches a minimum value. Thus stretch-induced alignment is sensitive to stretch frequency over a range of two orders of magnitude, with the actual values of the threshold and saturation frequencies dependent on the value of *τ*.

### Model Predictions

**Figure 4:**
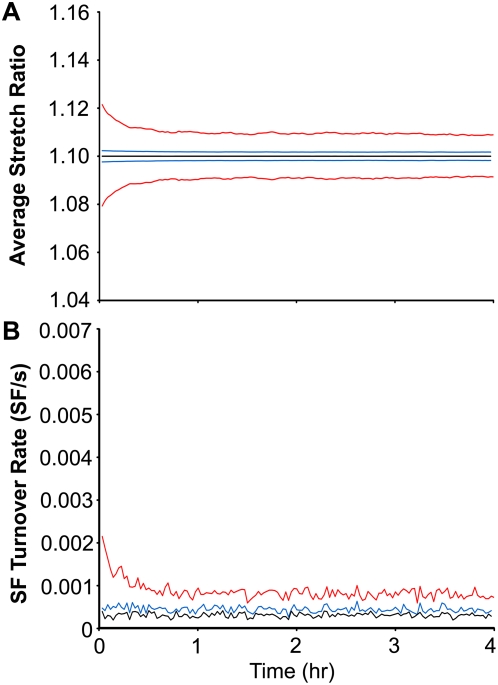
Predicted time evolutions of SF stretch and turnover rate in response to different frequencies of uniaxial stretch. The maximum and minimum values of the population-averaged fiber stretch during a cycle (A) and the rate of SF turnover (B) are shown for simulations of 10% cyclic uniaxial stretch at frequencies of 1 (red), 0.1 (blue) and 0.01 Hz (black) using the optimized parameter values.

In the absence of SF turnover and self-adjustment, the instantaneous population-average fiber stretch (*α*_avg_) would be expected to oscillate between the basal fiber stretch (*α*_0_ = 1.10) and a maximal value (1.155) corresponding to the fully deformed state of matrix stretch. When SF turnover and self-adjustment are considered, then *α*_avg_ changes over time (Figure 4A). For cyclic uniaxial stretch at 1 Hz, *α*_avg_ indeed oscillates between 1.10 and 1.155; however, within seconds, the maximum and minimum values for *α*_avg_ drop to 1.078 and 1.122 so that the time-averaged value of *α*_avg_ is equal to *α*_0_. This initial change in fiber stretch occurs before any fibers disassemble and reassemble and is thus completely attributable to SF self-adjustment, which causes the time-average value of each individual fiber to approach *α*_0_ with a characteristic time *τ*. While the time-averaged fiber stretch decreases almost immediately, the ratio of the maximum and minimum fiber stretch (i.e. the amplitude of fiber stretch) only slowly decreases. The slow decrease in the amplitude of fiber stretch occurs as a result of the gradual redistribution of SFs into orientations directed towards the direction of least perturbation in normal matrix strain (i.e. perpendicular to the direction of cyclic stretch). This alignment occurs because the fibers experiencing the greatest amplitudes of stretch (i.e. the orientation of large matrix normal strain) disassemble relatively quickly, leading to the accumulation of fibers in the direction of smallest matrix normal strain. By comparing the three curves in Figure 4A, it is clear that the amplitude of fiber stretch depends on stretch frequency. This frequency dependence occurs because the SFs dissipate more strain when strain rates are relatively small. At 0.01 Hz, the SF stretch does not vary despite the fact that the matrix is stretching. Without asymmetry in SF stretch, there is no stimulus for SF alignment (cf. Figure 2 for 0.01 Hz).

The rate constant for fiber disassembly is proportional to the deviation of fiber stretch from the equilibrium value *α*_0_. Consequently, the rate of SF turnover is highest when the deviation of fiber stretch is highest, which is immediately after initiating cyclic stretch (Figure 4B). For 1 Hz stretch, the initial drop in time-averaged *α*_avg_ results in a rapid decrease in SF turnover rate. The turnover rate continues to decline over the next 2 hours as the amplitude of fiber stretch decreases due to the gradual orientation of fibers perpendicular to the direction of stretch. For 0.01 Hz stretch, the rate for SF turnover remains at the basal level at all times, while an intermediate response is observed at 0.1 Hz.

### Effect of cyclic equibiaxial stretch

**Figure 5:**
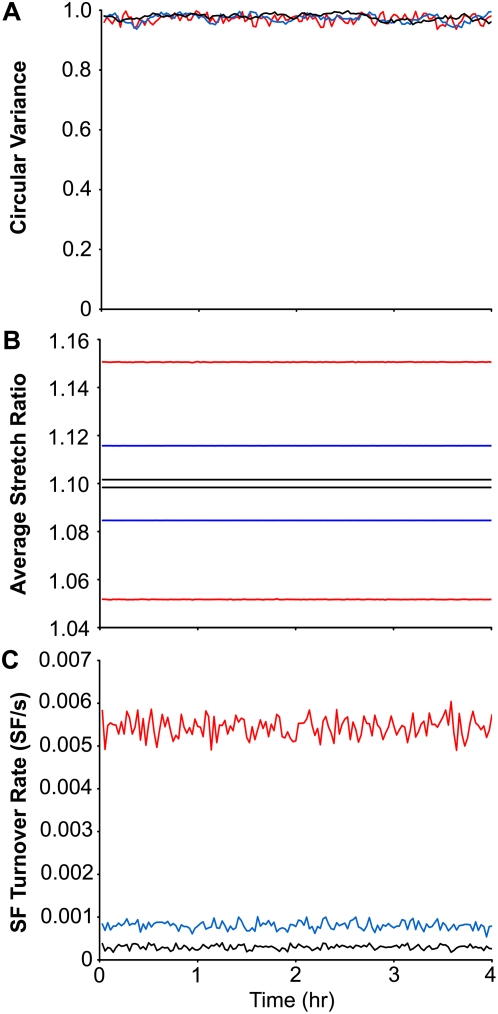
Predicted time evolutions of circular variance, SF stretch and fiber turnover rate in response to different frequencies of equibiaxial stretch. The circular variance (A), the maximum and minimum values of the population-averaged fiber stretch during a cycle (B), and the rate of stress fiber turnover (C) are shown for simulations of 10% cyclic equibiaxial stretch at frequencies of 1 (red), 0.1 (blue) and 0.01 Hz (black) using the optimized parameter values.

In contrast to cyclic uniaxial stretch, cyclic equibiaxial stretch of ECs does not induce SF alignment [8], [9]. Similarly, simulations of cyclic equibiaxial stretch do not result in the preferred orientation of SFs in any particular direction at any frequency (Figure 5A) since there is no direction of minimum matrix normal strain. The average fiber stretch is dependent on stretch frequency (Figure 5B), resulting in smaller fiber stretch amplitudes at lower stretch frequencies. Since SFs do not reduce their stretch by orienting perpendicular to the direction of stretch, the amplitude of fiber stretch remains elevated for stretch frequencies of 0.1 and 1 Hz (Compare Figures 4A and 5B). Similarly, there is a sustained elevation in SF turnover rates that is dependent on the frequency of stretch (Figure 5C). Thus, while there is no difference in stress fiber orientation, our model predicts that stress fibers will respond differently to cyclic equibiaxial stretches at different frequencies.

### Effect of uniaxial stretch magnitude

**Figure 6:**
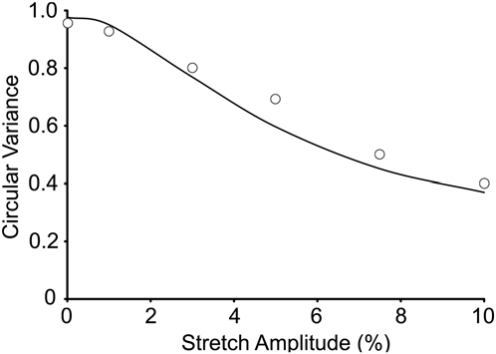
Comparison between measurements and model predictions of effect of cyclic uniaxial stretch magnitude on SF alignment. Simulations of 6 hr of cyclic uniaxial stretch at 1 Hz were performed over stretch magnitudes of 0 (static control) to 10% and the circular variances of the SF distributions were determined using the optimized parameter values. Circular variances of experimentally measured SF distributions (published in Kaunas et al. [17]) for cyclic uniaxial stretch at 1 Hz of non-confluent bovine aortic ECs transfected with Green Fluorescent Protein (circles) are shown for comparison.

We have previously reported the relationship between SF alignment and the magnitude of cyclic uniaxial stretch at 1 Hz [17]. These experiments were simulated using the optimized parameters estimated above (cf. Figure 2A). The model predicts a similar relationship between the steady-state circular variance and stretch magnitude as the experimental measurements (Figure 6). It should be noted that the model would provide a significantly better fit if the model parameters were optimized for this set of data. By using the parameters optimized from the other data set (cf. Figure 2), these results illustrate the ability of the model to predict the effects of stretch magnitude on SF alignment.

## Discussion

Cells in the body are continuously subjected to mechanical strains and respond through both structural and biochemical changes [19]. Often, stretching stimulates the activation of intracellular signals and the subsequent induction of genes that contribute to pathological situations such as atherosclerosis [20]–[23]. When cells are subjected to cyclic stretch in vitro, the activation of these pathophysiological signaling events is often transient, indicating that cells are able to adjust to the cyclic stretching in order to maintain biochemical homeostasis. The ECs in straight, unbranched arteries are subjected to cyclic stretch, yet maintain an anti-atherogenic phenotype [24], suggesting that the ECs have found a way to adapt. ECs in these straight, unbranched arteries are oriented perpendicular to the principal direction of stretch, while ECs do not align at arterial branches, where atherosclerosis typically occurs. Thus, cell alignment appears to contribute to the ability of ECs to maintain homeostasis in their dynamic mechanical environment. The present study quantifies the contributions of two mechanisms by which ECs and their SFs can adapt to cyclic stretch - SF reorientation and self-adjustment of their extension.

Our mathematical model describes the time-dependent changes in SF extension and orientation in terms of the rates of the turnover of SFs and the re-establishment of SF extension. The results of stretch experiments performed at different stretch frequencies were used to estimate physiologically relevant values for the model parameters. The model predicts that the extent of SF alignment depends on the whether SFs can self-adjust their level of extension faster than the SFs are stretched. Specifically, there is a threshold frequency of ∼0.01 Hz, below which SFs are able to self-adjust in order to maintain SF stretch at a set-point value. As the frequency of stretch increases from 0.01 to 1 Hz, the cells become increasingly less capable of adjusting to matrix stretch-induced changes in SF stretch. To compensate, SFs become increasingly more likely to orient toward the direction of least perturbation in stretch (i.e. perpendicular to the direction of stretch). The model also predicts that there is an upper threshold frequency of ∼1 Hz stretch where near-maximal SF alignment occurs.

There are few reports examining the effect of stretch frequency on stretch-induced cell alignment. Wille et al. [25] examined the effects of 10% cyclic uniaxial stretch at frequencies ranging from 0.25 to 1 Hz on the rate and extent of alignment of non-confluent human aortic ECs and concluded that the results were insensitive to stretch frequency. Liu et al. [26] tested the effects of stretch frequencies ranging from 0.5 to 2 Hz in vascular smooth muscle cells and reported a small, but significant, effect on cell alignment in which the greatest alignment was observed at 0.5 Hz. More recently, Jungbauer et al. [27] studied the effects of a wide range of stretch frequencies (0.0001 to 20 Hz) and demonstrated that both the rate and extent of fibroblast alignment was highly dependent on the frequency of stretch. We and others have quantified SF alignment in response to cyclic stretch [9], [17], [28]; however, the present study is the first to quantify the effects of stretch frequency on SF alignment. Quantitative data regarding stretch-induced SF alignment is critical for the development and testing of computational models of stretch-induced SF reorganization.

A stochastic model is well-suited to predict the dynamic changes in a population of SFs. Our experiments indicate that the population-average orientation of SFs in ECs is always perpendicular to the direction of stretch; however, not all the SFs are oriented in the same direction. The dispersion in SF orientation is a consequence of the disproportionate disassembly of SF in directions with higher normal matrix strains as well as the randomized direction of SF reassembly. Specifically, the number of SFs oriented in a particular direction is proportional to the expected lifetime of SFs in that particular direction. The lifetime of a SF is inversely related to the level of perturbation in SF stretch, hence SF tend to orient away from the direction of stretch. For equibiaxial stretch, the expected lifetime of a SF is the same in all directions, so the distribution of SF orientation remains relatively uniform (cf., Figure 5A). The model does predict that the amplitude in SF stretch and the rate of SF turnover are each proportional to the frequency of equibiaxial stretch (cf., Figs. 5B and C). Since SF assembly is assumed to occur immediately after disassembly, equibiaxial stretch does not lead to a change in the total number of SFs.

The present model of SF reorientation in response to cyclic matrix stretch shares some key features of a recent model proposed by De et al. [11]. In their deterministic model, cells readjust their contractile activity in an attempt to maintain either the local stress or strain in the surrounding matrix at a set-point value while being subjected to a periodic external stress [12]. At low frequencies of cyclic stress, the cells are able to readjust their contractile activity so as to maintain the mechanical state of the matrix at the set-point value and the cells orient parallel to the direction of stress, while at high frequencies the cells orient nearly perpendicular to the applied stress since the contractile activity is too slow to compensate. In the present stochastic model in which a periodic stretch is applied, SFs attempt to maintain constant the level of strain in the stress fibers, not in the surrounding matrix. At low stretch frequencies, the SFs are able to readjust their extension so as to maintain stretch at the set-point level with the result that the SFs do not orient in any direction. The contrasting results at low frequencies is attributable to the difference in the boundary conditions – De et al. [11] use traction boundary conditions, while the present model uses displacement boundary conditions. When the displacement is sufficiently slow, the cells essentially no longer sense the changing boundary conditions, while a cell would be expected to continue to sense a static or quasistatic stress. In the case of cells subjected to cyclic stretch on elastomeric substrates such as silicone rubber, we submit that the displacement boundary condition is a more appropriate description of the mechanical stimulus that the cells respond to. This is consistent with the concept of “stress shielding” in which stress in tissues is primarily borne by the matrix and is not transmitted to the resident cells [29]. Traction boundary conditions are expected to be more important in matrices with relatively low elastic moduli such as collagen hydrogels [30]. Wei et al. [31] proposed a dynamic model of cyclic stretch-induced SF orientation in which SF growth depends on the rate of SF shortening. In their model, cells initially devoid of SFs begin to assemble SFs under cyclic stretch conditions; with the rate of SF growth being greatest in the directions with least matrix shortening such as occurs during the retraction phase of a matrix stretch cycle. Their model also predicts that SF alignment depends on stretch magnitude and frequency, although the extent of alignment does not saturate at 1 Hz. It would be interesting to experimentally test the hypothesis that SF alignment is dependent on the rate of shortening by applying stretch waveforms in which the rate of rise and rate of fall of the matrix stretch unequal such that the rate of SF shortening is changed without changing the cycle period length. We are currently assembling a stretch device capable of generating such stretch waveforms.

Two characteristic times describe the SF response to cyclic stretch. The time constant for SF self-adjustment *τ* determines the sensitivity of SF to the frequency of stretch. When the characteristic time for self-adjustment is shorter than the period of a cycle of stretch, then the SFs can self-equilibrate to maintain SF extension at the set-point level. In contrast, the amplitude of SF extension follows that of the normal matrix strain when the period of the stretch cycle is much shorter than *τ*. The other characteristic time is that for SF disassembly (1/*k* ^i^), which depends on the level of perturbation of fiber stretch from the set-point level. For cyclic uniaxial stretch, the time constant for SF disassembly is much greater perpendicular vs. parallel to the direction of matrix stretch, leading to the accumulation of SFs in the perpendicular direction. The asymmetry in the time constants for disassembly decreases at low stretch frequencies since SF self-adjustment reduces the perturbation in SF extension from the set-point level. Consequently, the extent of SF alignment increases with frequency since the rate of stretching is faster than the rate of SF self-adjustment.

Parameter sensitivity analysis illustrates the effect of each model parameter on the system response. The rate and extent of SF alignment are primarily dependent on the values of *k*_0_ and *k*_1_, respectively. The rate of SF alignment in the present study is faster than the rate we reported for confluent ECs [13], thus the value of *k*_0_ is higher (3×10^−4^ vs. 10^−5^ s^−1^). This is consistent with the report that actin filament turnover is faster in non-confluent than confluent cells [32]. In addition, the extent of alignment in the present study is somewhat less than that for confluent ECs [13]. It is possible that when cells are confluent, cell-cell junctions may act to reinforce mutual alignment of neighboring cells and their SFs. Indeed, even in static cell culture we typically see localized co-alignment of SFs in small groups of cells, which is no longer apparent when considering larger populations of cells in the same culture. In the presence of a directional cue (i.e. uniaxial stretch), all cells tend to align in the same direction and the localized reinforcement may augment the uniformity of the SF alignment under confluent conditions.

The third model parameter, *τ*, primarily determines the frequency range over which circular variance transitions between the maximum and minimum values (i.e., the threshold and saturation values). We estimated a value of 0.5 s for τ, which described the measured transition of SF alignment from essentially no alignment at 0.01 Hz to extensive alignment at 1 Hz. Jungbauer et al. [27] did report a saturation frequency of 1 Hz for stretch-induced cell alignment, as well as exponential behavior similar to our own observations for SF alignment. We could not further increase stretch frequency with our system to test the prediction that 1 Hz is a saturation level of stretch frequency above which no additional alignment is expected. The time constant of 0.5 s is somewhat shorter than the value of ∼6 s obtained by Kumar et al. [18] from measured retraction rates of severed SFs in bovine capillary ECs. For cyclic stretch, SF self-adjustment occurs via SF lengthening during half the cycle and shortening during the other half of the cycle. It is possible that self-adjustment occurs at different rates depending on if the SF is lengthening or shortening, in which case the value of 0.5 s from the current study would represent an average of the two time constants. Another potential explanation is that cells from different vascular beds have different rates of self-adjustment. Arterial ECs are subjected to high frequency stretch (∼1–2 Hz), hence may need to be more responsive to time-changing strains than capillary ECs that experience much less frequent changes in matrix strain.

The current model is limited to describing a population of stress fibers and does not consider other factors that may interact with stress fibers during stretch-induced reorientation. Jungbauer et al. [27] reported that fibroblasts initially decrease their level of elongation during the first 10 minutes of cyclic stretch and then re-extend in the direction perpendicular to stretch. Cell elongation and stress fiber orientation are generally tightly coupled in highly polarized cells such as fibroblasts and smooth muscle cells and somewhat less so in more highly spread cells such as endothelial cells. Stress fiber assembly has been reported to drive endothelial cell elongation [33]. On the other hand, endothelial cell elongation has been proposed to drive the orientation of stress fibers [34]. In our model, new fibers are allowed to assemble in any direction with equal probability. It is likely that fiber assembly depends to some extent on cell shape, as well as the predominant orientations of the existing stress fiber population. These factors are expected to provide a virtual inertia for stretch-induced stress fiber reorientation, which may contribute to the ∼15 minute delay before the circular variance in stress fiber orientation began to decrease at a rate similar to that predicted for cyclic stretch at 1 Hz (cf. Fig. 2). SF assembly occurs immediately after a SF is disassembled in our model; however, SF assembly is a gradual process that involves actin polymerization and bundling, as well as focal adhesion assembly. The rates of these processes are expected to also influence the rate of stress fiber alignment. In future refinements of the current model we will investigate the relationships amongst stretch-induced changes in cell shape, SF and focal adhesion formation, and SF reorientation.

In conclusion, mathematical modeling provides a valuable tool for interpreting the strain rate-dependent SF reorganization induced by matrix stretching. When the rate of stretch is fast relative to the rate of SF self-adjustment, the SFs at non-equilibrium stretch values disassemble at relatively faster rates and gradually accumulate in directions of smallest perturbation from equilibrium. When the rate of stretch is slow, the SFs can self-equilibrate to maintain their level of extension, thus diminishing the mechanical signal for SF remodeling. While the current study focused on the effects of cyclic stretch on ECs, the model can be applied to SF reorganization in other cell types subjected to any two-dimensional matrix stretch pattern. We wish to emphasize that the present results only provide guidance toward the development of more realistic mathematical descriptions of SF remodeling. The phenomenological description of the self-adjustment of SF mechanical equilibrium needs to be supported with mechanistic details, such as the kinetics of actin-myosin crossbridging and α-actinin binding [2]. Nonetheless, the present results demonstrate the significance of SF self-adjustment in regulating mechanosensitivity, providing a mechanism for cells to cope with deformations of the tissues they reside upon.
